# Antioxidant Properties of Agri-Food Byproducts and Specific Boosting Effects of Hydrolytic Treatments

**DOI:** 10.3390/antiox9050438

**Published:** 2020-05-18

**Authors:** Federica Moccia, Sarai Agustin-Salazar, Luisella Verotta, Enrico Caneva, Samuele Giovando, Gerardino D’Errico, Lucia Panzella, Marco d’Ischia, Alessandra Napolitano

**Affiliations:** 1 Department of Chemical Sciences, University of Naples “Federico II”, Via Cintia 4, I-80126 Naples, Italy; federica.moccia@unina.it (F.M.); gderrico@unina.it (G.D.); dischia@unina.it (M.d.); alesnapo@unina.it (A.N.); 2 Departamento de Ingeniería Química y Metalurgía, Universidad de Sonora, Del Conocimiento, Centro, 83000 Hermosillo, Mexico; sarai.agustin@unison.mx; 3 Dipartimento di Scienze e Politiche Ambientali, Università degli Studi di Milano, Via G. Celoria 2, I-20133 Milan, Italy; luisella.verotta@unimi.it; 4 Unitech COSPECT, Direzione servizi per la Ricerca, Università degli Studi di Milano, Via C. Golgi 33, I-20133 Milan, Italy; enrico.caneva@unimi.it; 5 Centro Ricerche per la Chimica Fine Srl for Silvateam Spa, Via Torre 7, I-12080 San Michele Mondovì, CN, Italy; sgiovando@silvateam.com; 6 CSGI—Consorzio Sistemi a Grande Interfase, Department of Chemistry, University of Florence, Via della Lastruccia 3, I-50019 Sesto Fiorentino (FI), Italy

**Keywords:** agri-food waste, antioxidant, tannins, lignin, acid hydrolysis, DPPH assay, FRAP assay, phenolic polymers, electron paramagnetic resonance (EPR)

## Abstract

Largely produced agri-food byproducts represent a sustainable and easily available source of phenolic compounds, such as lignins and tannins, endowed with potent antioxidant properties. We report herein the characterization of the antioxidant properties of nine plant-derived byproducts. 2,2-Diphenyl-1-picrylhydrazyl (DPPH) and ferric reducing/antioxidant power (FRAP) assays indicated the superior activity of pomegranate peels and seeds, grape pomace and pecan nut shell. An increase in the antioxidant potency was observed for most of the waste materials following a hydrolytic treatment, with the exception of the condensed tannin-rich pecan nut shell and grape pomace. UV-Vis and HPLC investigation of the soluble fractions coupled with the results from IR analysis and chemical degradation approaches on the whole materials allowed to conclude that the improvement of the antioxidant properties was due not only to removal of non-active components (mainly carbohydrates), but also to structural modifications of the phenolic compounds. Parallel experiments run on natural and bioinspired model phenolic polymers suggested that these structural modifications positively impacted on the antioxidant properties of lignins and hydrolyzable tannins, whereas significant degradation of condensed tannin moieties occurred, likely responsible for the lowering of the reducing power observed for grape pomace and pecan nut shell. These results open new perspectives toward the exploitation and manipulation of agri-food byproducts for application as antioxidant additives in functional materials.

## 1. Introduction 

Roughly one-third of food produced for human consumption, which is about 1.3 billion tons per year, is lost or wasted as the result of several processes occurring along the supply chain [1,2]. The agri-food industry in particular is responsible for the generation of high volumes of organic waste. Examples of the significant amount of fruit waste produced globally per year include 9 million tons of grape byproducts [3], 15 million tons of citrus waste [3] and 3 to 4.2 million tons from apple [4,5]. Additionally, banana processing generates about 9 million tons of waste per year [4,5], whereas pineapple waste (peel and core) represent approximately 59% of the raw material [6]. Other remarkable examples are pomegranate, whose byproduct worldwide production is estimated about 9 tons per 1 ton of juice [3], and pecan nut, of which 40–50% is usually discarded, with approximately 420,000 tons of nut shell produced worldwide every year [7]. Vegetable processing also produces huge amounts of wastes, particularly in the case of potato and tomato with a globally production of about 12 million tons [8] and more than 3 million tons of byproducts per year in Europe [9], respectively.

Disposal of these by-products represents a cost to the food processor and has a negative impact on the environment. On the other hand, these materials can be regarded as a largely available, low cost source not only of energy for biofuel production, but also of value-added compounds, whose recovery represents therefore a valuable opportunity [10,11]. In this context, a prominent role is occupied by phenolic compounds, which are well-known for their beneficial effects on human health, in the prevention of inflammation and oxidative stress-related chronic diseases [12,13,14,15,16,17]. These properties have been ascribed in part to their ability to act as potent antioxidants and scavengers of free radicals through hydrogen atom/electron donation or metal ion chelation [18,19,20], which have prompted their use not only as food supplements [15,21,22,23], but also as additives for functionalization of materials to be used, e.g., in biomedicine [12,24,25,26], cosmetic [27,28,29,30] or food industry [31,32,33,34,35].

On this basis, and given the wide distribution of natural phenolic compounds in agri-food wastes [3,11,12,36,37,38,39,40], these latter have been the focus of intense research work aimed at characterization of their properties for exploitation in several application areas [41,42,43]. In particular, agri-food byproducts rich in phenolic polymers such as tannins and lignins endowed with potent antioxidant and/or antimicrobial activity [12,44,45,46,47,48,49,50], have received considerable attention as active components, e.g., in cosmetics [51], biomedicine [52,53], food packaging [35,54,55,56,57,58] or for polymer stabilization and functionalization [59,60,61,62,63,64,65]. Recently, an expedient acid hydrolysis protocol for agri-food wastes involving treatment with concentrated HCl at 100 °C [66,67] has been developed in order to obtain materials with improved antioxidant properties. As a remarkable example, acid hydrolysis of spent coffee grounds led to a material with an antioxidant efficiency up to 30 times higher than that of the starting sample [68], exhibiting promising properties as an active component in food packaging or as a food supplement endowed with prebiotic activity and low cytotoxicity [69]. An increase of the antioxidant potency has been observed also in the case of pomegranate wastes [70] and exhausted woods from tannin extraction [49]. At a molecular level, the effects of the hydrolytic treatment have been interpreted in terms of removal of the polysaccharidic fraction and dehydration/aromatization of the phenolic components, making the OH functionalities more available for interaction with oxidized species [68].

Based on these findings, we report herein the results of a systematic evaluation of the antioxidant properties of a series of plant-derived byproducts, selected among those produced in largest amounts by the agri-food industry. The effects of the previously reported hydrolytic treatment on the antioxidant properties of the selected materials were also evaluated. In order to interpret the different activation/deactivation effects observed, the main structural modifications induced on the phenolic components by the hydrolytic treatment were finally investigated by spectroscopic techniques and chemical degradation methods, in comparison with those occurring on model natural and bioinspired, synthetic phenolic polymers.

## 2. Materials and Methods 

### 2.1. General Experimental Methods

Pomegranates, apples, oranges, bananas, pineapples, tomatoes and potatoes were purchased at a local supermarket. Chestnut and quebracho tannins were provided by Silvateam (S. Michele Mondovì, Cuneo, Italy). Grape pomace was kindly provided by Dott. Daniele Naviglio (Department of Chemical Sciences, University of Naples “Federico II”, Naples, Italy). Pecan nut shell was provided by Productora de Nuez S.P.R. de R.I. (Hermosillo, Mexico). Horseradish peroxidase (HRP), hydrogen peroxide 30%, 2,2-diphenyl-1-picrylhydrazyl (DPPH), iron (III) chloride (97%), 2,4,6-tris(2-pirydyl)-s-triazine (TPTZ) (≥98%), Folin-Ciocalteu reagent, (±)-6-hydroxy-2,5,7,8-tetramethylchromane-2-carboxylic acid (Trolox) (97%), gallic acid (≥97.5%), ellagic acid, caffeic acid, ferulic acid, vanillic acid, 4-hydroxybenzoic acid (4-HBA) and 3,4-dihydroxybenzoic acid (3,4-DHBA) were obtained from Sigma-Aldrich (Milan, Italy). 

UV-Vis spectra were recorded using a HewlettPackard 8453 Agilent spectrophotometer. Attenuated total reflectance-Fourier transform infrared (ATR-FTIR) spectra were recorded on a PerkinElmer Spectrum 100 spectrometer, equipped with a Universal ATR diamond crystal accessory. Spectra were recorded as an average of 16 scans in the range 4000−450 cm^−1^ (resolution of 4 cm^−1^). 

HPLC analysis was performed with an instrument equipped with a UV-Vis detector (Agilent, G1314A); a Phenomenex Sphereclone ODS column (250 × 4.60 mm, 5 µm) was used, at a flow rate of 1.0 mL/min; a 0.1% formic acid (solvent A)/methanol (solvent B) gradient elution was performed as follows: 5% B, 0–10 min; from 5 to 80% B, 10–57.5 min; the detection wavelength was 254 nm. The inter-day and intra-day precision exhibited a coefficient of variation <3% (*n* = 40) over 1 week and <8% over 1 month on the same column. Accuracy ranged from 98% (*n* = 40 over 1 week) to 95% over 1 month.

Electron paramagnetic resonance (EPR) measurements were performed using a Bruker Elexys E-500 spectrometer equipped with a superhigh sensitivity probe head. The samples were transferred to flame-sealed glass capillaries, which in turn were coaxially inserted in a standard 4 mm quartz sample tube. Measurements were performed at room temperature. The instrumental settings were as follows: sweep width, 100 G; resolution, 1024 points; modulation amplitude, 1.0 G; scansion time 20.97 s. The amplitude of the field modulation was preventively checked to be low enough to avoid detectable signal overmodulation. The number of scans and microwave power were optimized to avoid microwave saturation of resonance absorption curve. For power saturation experiments, the microwave power was gradually incremented from 0.02 to 164 mW. The *g* value and the spin density were evaluated by means of an internal standard, Mn^2+^-doped MgO, prepared by a synthesis protocol reported in the literature [71]. Since sample hydration was not controlled during the measurements, spin density values have to be considered as order of magnitude estimates [72].

Solid-state ^13^C cross-polarization magic angle spinning (CP-MAS) spectra were collected at 125.77 MHz on a 500 MHz Bruker BioSpin NMR Spectrometer Avance 500, operating at a static field of 11.7 T and equipped with a 4 mm MAS probe, spinning the sample at the magic angle at speeds up to 15 kHz that with the addition of high power ^1^H decoupling capability allows to decrease or eliminate homo and heteronuclear anisotropies. All the samples were prepared by packing them in zirconia (ZrO_2_) rotors, closed with Kel-F caps (50 μL internal volume) and the spinning speed (MAS) was optimized at 12 kHz after some experiments run in the range 4−12 kHz. Cross-polarization (CP) spectra under Hartmann−Hahn conditions were recorded with a variable spin-lock sequence (ramp CP-MAS) and a relaxation delay of 4 s; a ^1^H π/2 pulse width of 3.0 μs was employed. Contact time was varied in the range 1−2.5 ms. In some experiments, high power proton decoupling was applied during acquisition without cross-polarization. CP was also run with non-quaternary suppression experiment, rotor synchronized “NQS” refocused (CPNQS), to study the intensity of different heteronuclear dipolar interactions. In addition to CPNQS experiments, CP editing experiment by phase inversion (CPPI) was also used, as comparison, analyzing the evolution of dephasing ^13^C signal, depending on the heteronuclear dipolar interaction intensities [73,74].

### 2.2. Preparation of Agri-Food Byproducts

The waste part of each agri-food product was separated from the edible part, rapidly cut into small pieces (175 g for pomegranate, 235 g for apple, 195 g for orange, 99 g for banana, 308 g for pineapple, 95 g for potato and 333 g for tomato) and freeze-dried. The lyophilized material (97 g for pomegranate, 132 g for apple, 130 g for orange, 41 g for banana, 47 g for pineapple, 81 g for potato and 19 g for tomato) was finally shredded using a blender.

### 2.3. Preparation of Polymers from Caffeic Acid (PolyCAF) and Ferulic Acid (PolyFER)

A procedure previously reported was adopted [75]. Briefly, 200 mg of caffeic or ferulic acid was solubilized in ethanol and added to 0.1 M phosphate buffer (pH 6.8) containing 1% KCl (10 mM final concentration of the compound) (ethanol/buffer ratio = 1:4 *v*/*v*). Horseradish peroxidases (2 U/mL final concentration) and 30% H_2_O_2_ (20 mM final concentration) were then added in two aliquots at 1 h interval. The mixture was kept under magnetic stirring overnight, acidified with 3 M HCl up to pH 3 and kept at 4 °C for 24 h. The precipitate was then recovered by centrifugation (8247× *g*, 4 °C, 30 min), washed with 0.1 M HCl, and recovered by lyophilization.

### 2.4. Hydrolytic Treatment

The waste materials or the tannin samples (3 g) were treated with 70 mL of 6 M HCl under stirring at 100 °C for 1 h [49,68,70]. After cooling at room temperature, the mixture was centrifuged (8247× *g*, room temperature, 15 min) and the precipitate washed with water until neutrality and freeze dried. The recovery yields (*w*/*w*) for tannins were 67% (quebracho tannins) and 41% (chestnut tannins). The same procedure was applied also to PolyCAF and PolyFER but using 100 mg of the starting material. The recovery yields (*w*/*w*) were 83% (PolyCAF) and 67% (PolyFER).

### 2.5. DPPH Assay

To a 0.2 mM ethanolic solution of DPPH, the different agri-food byproduct or tannin powders, before and after hydrolytic treatment, were added (final dose 0.05−4.5 mg/mL), and after 10 min under stirring at room temperature, the absorbance of the solution at 515 nm was measured. In the case of PolyCAF and PolyFER, 30–400 µL of a 0.33 mg/mL polymer solution in DMSO were added to 2 mL of the DPPH solution and the mixtures were analyzed as above. Trolox was used as a reference antioxidant. Experiments were run in triplicate [76,77].

### 2.6. Ferric Reducing/Antioxidant Power (FRAP) Assay

To 0.3 M acetate buffer (pH 3.6) containing 1.7 mM FeCl_3_ and 0.83 mM TPTZ, agri-food byproduct and tannin powders, before and after hydrolytic treatment, were added (final dose 0.00625–0.3 mg/mL) and after 10 min under stirring at room temperature the absorbance of the solution at 593 nm was measured. In the case of PolyCAF and PolyFER, 5–500 µL of a 0.33 mg/mL polymer solution in DMSO were added to 3.6 mL of the FRAP solution and the mixtures were analyzed as above. Results were expressed as Trolox equivalents (eqs). Experiments were run in triplicate [78].

### 2.7. Total Phenolic Content (TPC) Assay

The different agri-food byproducts or tannins, before and after hydrolytic treatment, were added at a final dose of 0.02−3 mg/mL to a solution consisting of Folin-Ciocalteu reagent, 75 g/L Na_2_CO_3_, and water in a 1:3:14 *v*/*v*/*v* ratio. After 30 min incubation at 40 °C, the absorbance at 765 nm was measured. Gallic acid was used as reference compound. Experiments were run in triplicate [79]. 

### 2.8. Alkali Fusion

Solid and finely grinded KOH (100 mg), NaOH (100 mg) and Na_2_S_2_O_4_ (2 mg) were mixed in a pyrex tube and kept at 240 °C until fusion of the reagents. Then, 20 mg of each waste, before and after hydrolytic treatment, was added and the mixture was kept at 240 °C for additional 10 min. After cooling to room temperature, 10 mL of a 1% sodium dithionite (Na_2_S_2_O_4_) solution were added. After addition of 6 M HCl, the mixture was extracted with ethyl acetate (3 × 15 mL) and the combined organic layers were dried over sodium sulfate and evaporated to dryness. The solid residue was reconstituted in 2 mL of methanol, cleared by filtration on a 0.45 µm polyvinylidene fluoride (PDVF) filter, and analyzed by HPLC [35,80]. 

### 2.9. Alkaline Hydrogen Peroxide Degradation

10 mg of each agri-food byproduct sample was suspended in 1 M NaOH (1 mL) and 50 µL of 30% H_2_O_2_ were added. The mixture was kept at room temperature under vigorous stirring and after 24 h treated with 5% w/v Na_2_S_2_O_5_, taken to pH 3 with 6 M HCl, filtered on a 0.45 µm PVDF filter, and analyzed by HPLC [66].

### 2.10. Acid Degradation

50 mg of each agri-food byproduct was placed in a pyrex tube. Then, 5 mL of 4 M HCl was added and the mixture was vortexed for 1 min and incubated in an oven at 90 °C for 24 h. The samples were then allowed to cool to room temperature and the pH was adjusted to 2.5 with 6 M NaOH, after that they were centrifuged for 10 min at 4123× *g*. The supernatants were recovered, taken to 10 mL by addition of water, and analyzed by HPLC after filtration on a 0.45 µm PVDF filter. The solid residues were dissolved in 10 mL of DMSO/methanol 1:1 *v*/*v* and then analyzed by HPLC as well [81]. 

### 2.11. Extraction of Phenolic Compounds

10 mg of each agri-food byproduct or tannin (before and after hydrolytic treatment) was added to 1 mL of DMSO. The mixtures were kept under magnetic stirring at room temperature for 1 h, and then centrifuged (3534× *g*., 20 min). The supernatants were diluted in methanol (1:50 or 1:500 *v*/*v*) and analyzed by HPLC and UV-Vis spectrophotometry. 

## 3. Results and Discussion 

### 3.1. Antioxidant Properties of the Agri-Food Byproducts

The antioxidant properties of the selected agri-food byproducts were investigated by two widely used assays, i.e., the DPPH and FRAP assay, following the “QUENCHER” method, which allows to measure the efficiency of electron transfer processes from a solid antioxidant [76,77,78]. The results are reported in Table 1, together with data obtained for the reference antioxidant Trolox. The lowest EC_50_ values in the DPPH assay were determined for pecan nut shell, pomegranate peel and seeds and grape pomace, in that order, being significantly more active than the other byproducts tested, which exhibited EC_50_ values higher than 1.5 mg/mL. A similar trend was observed in the FRAP assay, with banana and pineapple wastes characterized by a number of Trolox eqs circa one order of magnitude lower than those exhibited by pecan nut shell, pomegranate wastes and grape pomace, whereas the other agri-food byproducts showed an even lower reducing activity.

These marked differences in the antioxidant properties may be likely interpreted considering the kind and the amount of phenolic compounds present in the different agri-food byproducts. To obtain information in this regard, the TPC was determined for each sample by the Folin-Ciocalteu assay [79] (Table 1). As expected, the highest TPC values were determined for pomegranate peel and seeds, grape pomace and pecan nut shell, although no correlation with the Trolox eqs determined in the FRAP assay or the EC_50_ values measured in the DPPH assay was found (data not shown). This would suggest that other factors (e.g., type of phenols or relative solubility in the assay medium) might affect the antioxidant efficiency of the materials. For example, pomegranate wastes are rich in hydrolyzable ellagitannins such as punicalagin and punicalin, which are endowed with very high antioxidant potency and characterized by high water solubility [70,82]. Condensed tannins (proanthocyanidins) are instead the main phenolic components, together with small phenolic acids and anthocyanins, of grape pomace, which contains also relative high amounts of lignins (17–24%), whose insolubility in the majority of solvents is well-documented [11,38]. The same applies to other lignocellulosic materials such as pecan nut shell [35,60]. As to the less active agri-food byproducts, most of them, such as apple [38,83,84], potato [38,85] and banana peels [11,38], are characterized by the presence of flavonoids, (epi)catechins and chlorogenic acids, which, though able to exert powerful antioxidant activities in vitro and in vivo, are highly susceptible to oxidation by polyphenoloxidases, as evident from the rapid browning of these materials when exposed to air, with consequent loss of the antioxidant efficiency [86,87]. 

### 3.2. Effects of the Hydrolytic Treatment on the Antioxidant Properties of the Agri-Food Byproducts

The hydrolytic treatment on the agri-food waste was performed according to the protocol developed in previous studies [49,66,68,70], that is, by incubating them in 6 M HCl at 100 °C for 1 h. The hydrolyzed materials were then collected by centrifugation, extensively washed with water till neutrality, and freeze-dried to give a powder in variable yields as shown in Table 2. 

The higher yields obtained in the case of grape pomace and pecan nut shell can be attributed to the high content of phenolic polymers such as lignins and condensed tannins, which are insoluble in water and less easily hydrolyzable compared to starch and other polysaccharides abundant, e.g., in potato and apple byproducts. 

**Figure 1:**
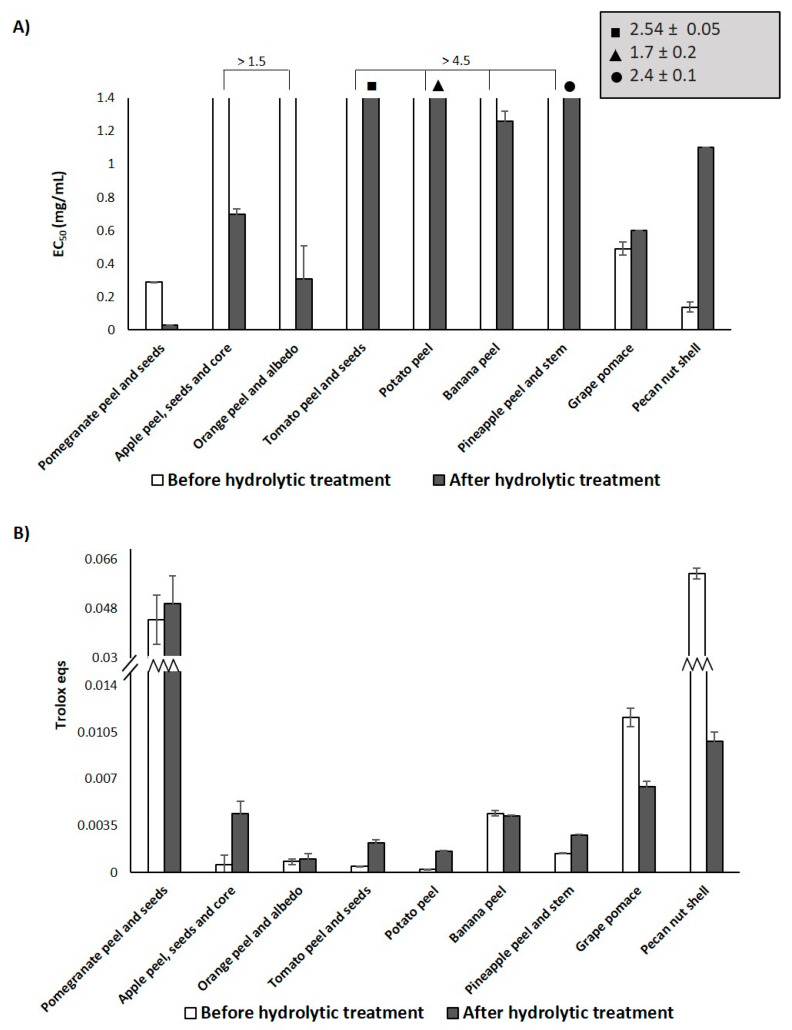
Antioxidant properties of agri-food byproducts before and after hydrolytic treatment. (**A**) DPPH assay; (**B**) FRAP assay. Reported are the mean ± SD values of at least three experiments.

The DPPH assay run on the hydrolyzed materials showed a remarkable ten-fold decrease of the EC_50_ value for pomegranate peel and seeds (Figure 1A). The activating effect of the hydrolytic treatment was also evident in the case of the other agri-food wastes, particularly those from apple, orange, tomato, potato, banana and pineapple (Figure 1A). By contrast, no significant improvement of the antioxidant properties was observed for grape pomace, while even a weakening in the DPPH-reducing properties was obtained in the case of pecan nut shell, exhibiting a circa eight-fold increase of the EC_50_ value further to the hydrolytic treatment.

The results of the FRAP assay (Figure 1B) showed that the hydrolytic treatment was again particularly effective in the case of apple, potato and tomato wastes, leading to an increase of the reducing capacity of about five–seven times, whereas the effects on orange, banana and pomegranate wastes were less significant. In perfect agreement with the DPPH assay results, the acidic treatment led to an 80% decrease of the Trolox eqs for pecan nut shell. A 50% decrease in the Trolox eqs was observed also in the case of grape pomace (Figure 1B).

**Figure 2:**
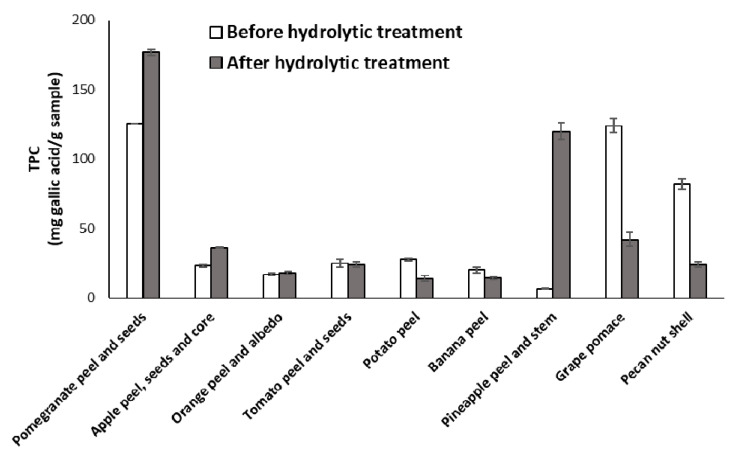
TPC of agri-food byproducts before and after hydrolytic treatment. Reported are the mean ± SD values of at least three experiments.

These results were partly in accordance with those obtained in the case of other agri-food byproducts such as spent coffee grounds or exhausted woods from tannin extraction [49,68], and could be interpreted as a consequence of the efficient removal of the hydrolyzable constituents, mainly carbohydrates, making the polyphenol-rich component more available for interaction with free radicals, as highlighted also by the generally low isolation yields of the hydrolyzed materials (Table 2). The Folin-Ciocalteu assay corroborated in part this hypothesis (Figure 2), since higher TPC values were determined, e.g., for pomegranate, apple and particularly pineapple wastes. Notably, TPC values about three-fold lower than those exhibited by the untreated materials were observed for hydrolyzed grape pomace and pecan nut shell, pointing to substantial chemical modifications induced by the hydrolytic treatment on these agri-food byproducts, also responsible for the lowering of the antioxidant properties emerging from the DPPH and the FRAP assay. Moreover, no correlations were again found between the antioxidant potency and the TPC values, suggesting that more complex transformations than the simple removal of the polysaccharidic fraction were induced by the hydrolytic treatment being responsible for the different effects observed. To investigate on this, the antioxidant properties of natural phenolic compounds and biomimetic phenolic polymers as models of the main phenolic compounds present in the selected agri-food wastes were evaluated before and after the same hydrolytic treatment performed on the agri-food byproducts. In addition, the chemical modifications induced on the agri-food waste by the hydrolytic treatment were investigated by spectroscopic and chemical degradation methods. 

### 3.3. Antioxidant Properties of Natural Tannins and Lignin-Mimicking Phenolic Polymers before and after the Hydrolytic Treatment

#### 3.3.1. Natural Tannins

**Figure 3:**
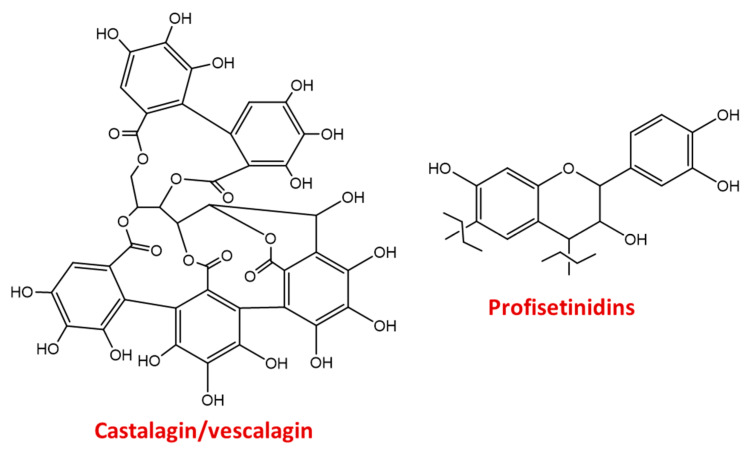
Representative structures of chestnut and quebracho tannins.

Based on their easy availability as industrial products, chestnut and quebracho tannins were chosen as models of the two main classes of tannins, that is hydrolyzable and condensed, respectively, present in the agri-food byproducts subject of the present study. Chestnut tannins are mainly composed of ellagitannins such as castalagin and vescalagin, whereas the major constituents of quebracho tannins have been identified as profisetinidins [12,49,88,89] (Figure 3). 

**Figure 4:**
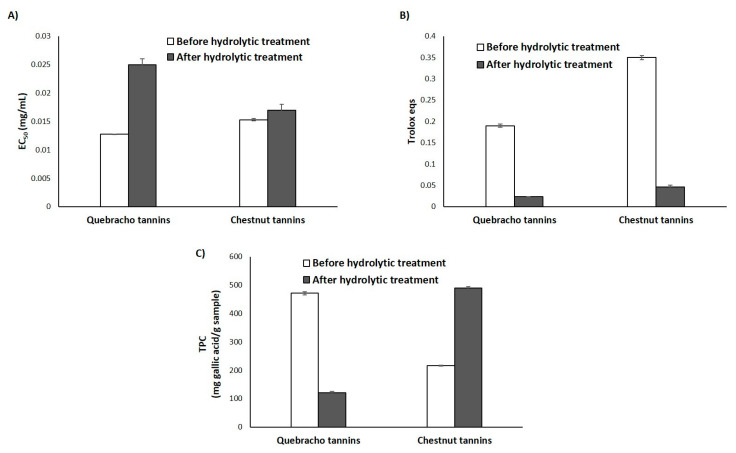
Antioxidant properties and TPC of quebracho and chestnut tannins before and after hydrolytic treatment. (**A**) DPPH assay; (**B**) FRAP assay; (**C**) Folin-Ciocalteu assay. Reported are the mean ± SD values of at least three experiments.

Figure 4 shows the results of the DPPH, FRAP and Folin-Ciocalteu assay obtained for the two tannin samples before and after the hydrolytic treatment. No differences were observed in the first case for chestnut tannins, whereas a decrease in the antioxidant properties further to the hydrolytic treatment was detected for quebracho tannins (Figure 4A), in line with what was observed for the agri-food byproducts rich in condensed tannins, that is grape pomace and pecan nut shell. A similar result was obtained in the FRAP assays, although in this case also the reducing properties of chestnut tannins were dramatically and negatively affected (Figure 4B). This data would reinforce the hypothesis that the effects of the hydrolytic treatment can not merely be attributed to the removal of the hydrolyzable components (e.g., sugar moieties) through glycosidic bond cleavage, but also to chemical modifications induced on the phenolic units. No correlation was observed, as before, between the TPC values (Figure 4C) and the antioxidant potency, since the hydrolytic treatment determined a decrease of the TPC value for the condensed quebracho tannins, but an increase, as expected, in the case of the hydrolyzable chestnut tannins, pointing again to the important contribution of several factors (e.g., solubility) as determinant of the antioxidant properties. 

#### 3.3.2. Lignin-Mimicking Phenolic Polymers

**Figure 5:**
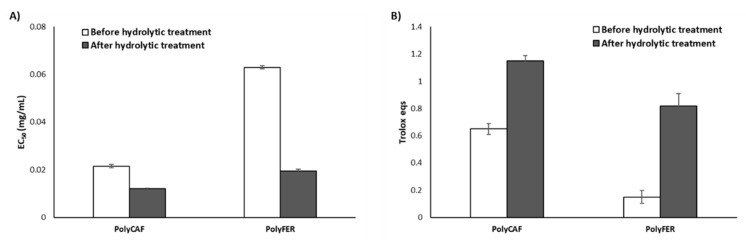
Antioxidant properties of PolyCAF and PolyFER before and after hydrolytic treatment. (**A**) DPPH assay; (**B**) FRAP assay. Reported are the mean ± SD values of at least three experiments.

Synthetic polymers obtained from HRP/hydrogen peroxide catalyzed oxidation of caffeic and ferulic acid (PolyCAF and PolyFER) were chosen as models of lignin [12,67,75] and subjected to the same hydrolytic treatment adopted for the agri-food byproducts. Both DPPH and FRAP assays indicated a remarkable increase in the antioxidant properties after the hydrolytic treatment, especially for PolyFER, which showed a circa 70% decrease of the EC_50_ value in the DPPH assay and a more than five-fold increase of the Trolox eqs in the FRAP assay (Figure 5). These data are in good agreement with those previously reported for lignin-rich agri-food byproducts, such as spent coffee grounds [68] or exhausted woods [49], showing a remarkable boosting of the antioxidant properties when subjected to the acid hydrolysis treatment. This effect was indeed attributed not only to the efficient removal of the non-antioxidant cellulose and hemicellulose fractions, but also to depolymerization-polymerization processes involving the lignin moieties amply documented in the literature [68,90,91,92].

To further address this issue, the structural modifications induced by the hydrolytic treatment on PolyCAF and PolyFER were examined by UV-Vis and EPR spectroscopy, and solid state ^13^C NMR. 

UV-Vis spectra of the acid-treated polymers (Figure S1) were characterized by a slight broadening of the absorption features, concerning particularly the band at 320 nm. 

**Figure 6:**
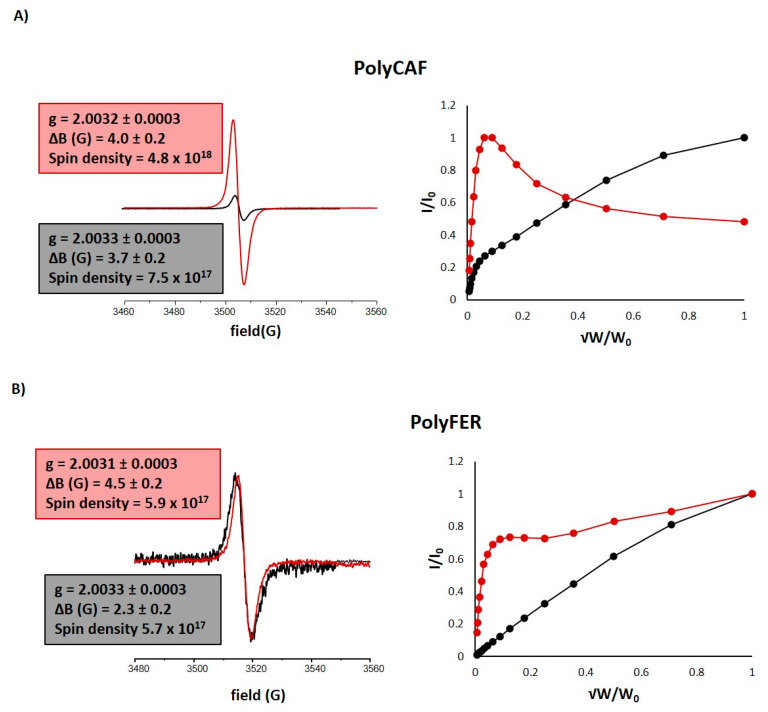
Solid state electron paramagnetic resonance (EPR) spectra and power saturation profiles of (**A**) PolyCAF and (**B**) PolyFER before (black traces) and after (red traces) hydrolytic treatment.

EPR spectroscopy has been recently proven to be a promising approach to inquire into the structural basis of the antioxidant activity of phenolic polymers, which are characterized by the presence of intrinsic free radical centers [75,93]. The EPR spectra of PolyCAF and PolyFER before and after the hydrolytic treatment are shown in Figure 6 along with the power saturation curves. The spectra of treated and untreated samples showed a similar line shape, with a single signal at a g value of ∼ 2.0033, consistent with the presence of carbon-centered radicals [72,75]. The weight normalized intensity was apparently much higher for the acid-treated PolyCAF (Figure 6A) compared to the starting sample, whereas no substantial variations were observed for PolyFER (Figure 6B). Quantitative determination of the signal amplitudes (ΔB) indicated broader signals for the polymers subjected to the hydrolytic treatment, especially in the case of PolyFER, likely indicating a higher variety of the free-radical population. Moreover, higher ΔB values could be taken as an indication of a larger number of π-stacking interactions resulting from a rearrangement of supramolecular aggregation as a consequence of the increase in aromatic (planar) units. This is in agreement with the relatively larger signal observed for π-stacked polymeric melanin pigments. Remarkable differences were also apparent from the normalized power saturation curves (Figure 6A,B), suggesting again that the hydrolytic treatment significantly affects the distribution and type of the intrinsic paramagnetic centers as a consequence of structural modifications induced in the phenolic polymers. 

Other valuable structural information was obtained by solid state ^13^C NMR run in the CP-MAS modality. 

**Figure 7:**
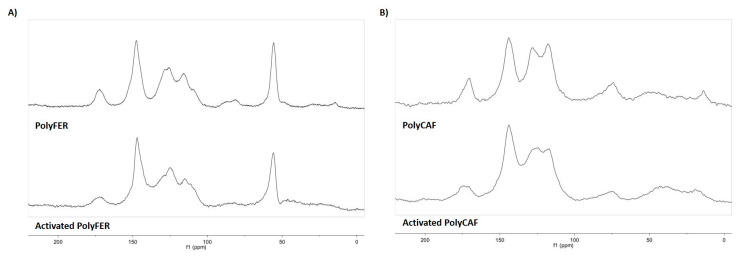
^13^C CP-MAS NMR of (**A**) PolyFER and (**B**) PolyCAF before (top) and after (bottom) hydrolytic treatment.

**Figure 8:**
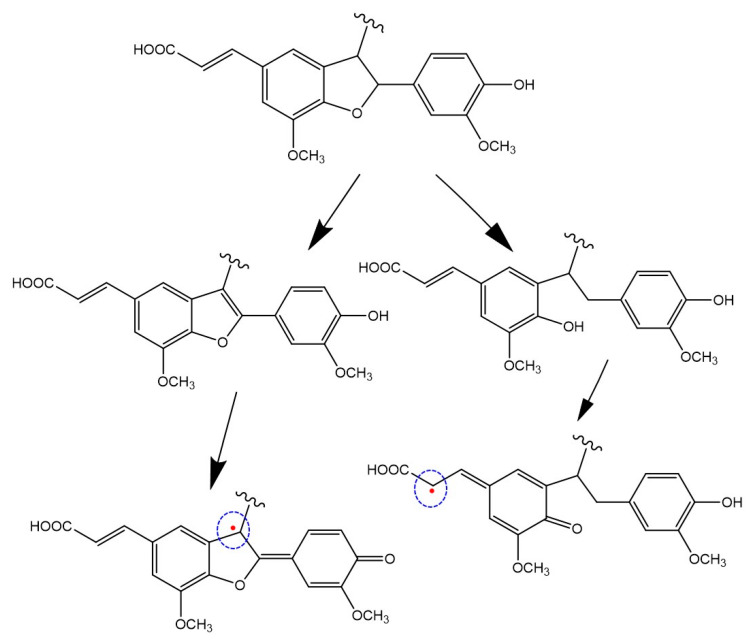
Proposed representative structural modifications and new paramagnetic centers induced on PolyFER by the hydrolytic treatment.

The spectrum of PolyFER (Figure 7A) featured broad signals around 172 and 56 ppm, due to carboxyl and methoxyl groups, respectively, and other signals around 150, 135–125, and 120–110 ppm. These latter were almost completely suppressed in the CP nonquaternary suppression (CP-NQS) spectrum (Figure S2), and hence, attributed to the C-H carbon of the benzene moiety, whereas the signals at 150 and 135–125 ppm, which were not substantially modified in the CP-NQS spectrum, could be assigned to quaternary aromatic carbons. These data were confirmed by the CP with polarization inversion (CPPI) spectrum (Figure S2). Another remarkable feature of the CP-MAS spectrum of PolyFER was the presence in the aliphatic region of weak but well discernible C-H carbon resonances in the range 75−90 ppm, indicative of the presence of dihydrobenzofuran units in agreement with what reported for structurally-related phenolic polymers [73,94] (Figure 8). A quite similar ^13^C NMR spectrum was recorded for PolyCAF (Figure 7B), although in this case the signal at circa 56 ppm was obviously lacking.

The major effect of the hydrolytic treatment on the ^13^C NMR spectra of the phenolic polymers was the almost complete disappearance of the broad signals at 75–90 ppm (Figure 7). A possible interpretation is that the treatment favors aromatization of the dihydrofuran units responsible for such signals and/or a number of phenolic functions are freed following the acid treatment, as exemplified in Figure 8 in the case of PolyFER. Both routes would lead to more extended π-electron conjugated species, that could be responsible for the increased antioxidant potency observed further to the hydrolytic treatment, as well as for the different paramagnetic properties highlighted by the EPR spectra [75].

### 3.4. Chemical Modifications Induced by the Hydrolytic Treatment on the Agri-Food Byproducts 

#### 3.4.1. ATR-FTIR Analysis

**Figure 9:**
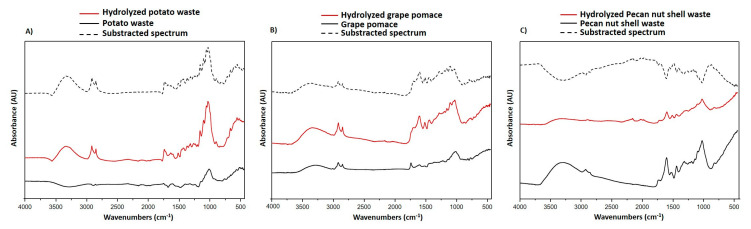
ATR-FTIR spectra of (**A**) potato waste, (**B**) grape pomace and (**C**) pecan nut shell.

To gain an insight into the processes that account for the observed potentiation or decrease of agri-food byproduct antioxidant properties, in subsequent experiments the structural modifications induced by the hydrolytic treatment were investigated by ATR-FTIR spectroscopy. As an example, the IR spectra of potato waste, pecan nut shell and grape pomace, before and after hydrolytic treatment, are shown in Figure 9. As evident also from the subtracted spectrum (hydrolyzed minus untreated), a marked increase of the two sharp peaks in the 2950–2850 cm^−1^ region, typically associated to the C−H stretching vibration of lignins [67,68,69], was observed further to the hydrolytic treatment in the case of potato waste (Figure 9A), thus indicating a significant enrichment in the lignin components likely responsible for the marked improvement of the antioxidant properties observed for this byproduct. In line with this interpretation, a substantial increase of the signals in the 2950–2850 cm^−1^ region was recorded also in the case of the other hydrolysis-activated materials such as orange, pineapple and especially tomato waste products (Figure S3). Notably, the peaks at 2950–2850 cm^−1^ were less significantly affected or even reduced by the hydrolytic protocol in the case of the agri-food byproducts whose antioxidant properties decreased further to this treatment, that is grape pomace and pecan nut shell (Figure 9B,C), strongly underlining the major role of lignin as determinants of the antioxidant properties of most plant-derived materials. 

#### 3.4.2. Analysis of the Extractable Fraction

To gain further information about the nature of the chemical modifications induced by the hydrolytic treatment on the agri-food byproducts, the DMSO-soluble fractions of the different materials were analyzed by UV-Vis spectroscopy and HPLC after proper dilution in methanol. DMSO was chosen as the solvent based on its ability to dissolve a wide range of most polar and non-polar natural phenolic compounds [81,95,96,97].

**Figure 10:**
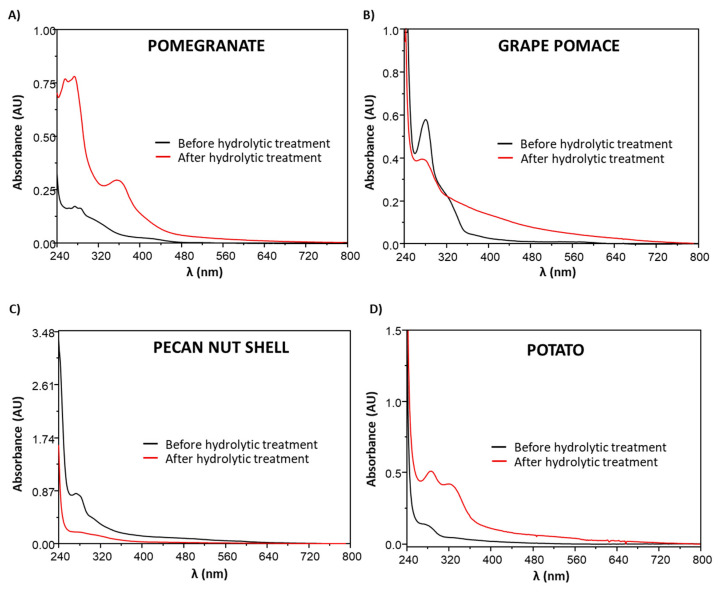
UV-Vis spectra of the DMSO extractable fraction of (**A**) pomegranate waste (0.02 mg/mL), (**B**) grape pomace (0.2 mg/mL), (**C**) pecan nut shell (0.2 mg/mL) and (**D**) potato waste (0.2 mg/mL) before and after the hydrolytic treatment. Concentrations refer to the starting dose of each agri-food waste in DMSO after proper dilution in methanol (see paragraph 2.11).

Generally, UV-Vis spectra showed an overall increase in absorbance after the hydrolytic treatment (Figure 10 and Figure S4), suggesting the efficient hydrolytic cleavage of phenolic polymers such as lignins and hydrolyzable tannins, giving rise to low-molecular weight, extractable compounds likely responsible for the increase in the antioxidant properties observed. This effect was particularly evident in the case of potato peels and pomegranate wastes (Figure 10A,D). In fact, the potato waste spectrum recorded after the acid treatment showed an increase in the absorption maxima at around 280 and 310 nm, typical of lignin moieties with hydroxycinnamic acid type structures [69,98,99] in agreement with what observed by the ATR-FTIR analysis; in the case of pomegranate peels and seeds, the UV-Vis spectrum of the acid-treated sample showed a maximum at 355 nm, suggestive of the presence of ellagic acid as a result of punicalagin and punicalin hydrolysis as already reported for pomegranate wastes [70,82] and confirmed by HPLC analysis of the DMSO-extractable fraction (Figure S5). Notably, similar results were observed in the case of chestnut tannins, selected as a model of hydrolyzable tannins as described above (Figure S6). Formation of ellagic acid would likely account for the increase of the antioxidant properties observed for pomegranate byproducts in the DPPH assay, given its higher solubility in alcoholic solvents compared to the water-soluble ellagitannins; on the other hand, the low solubility of ellagic acid in aqueous media would be in line with the not substantial improvement of the reducing properties determined in the FRAP assay (Figure 1).

Unlike what was observed for the majority of agri-food wastes, grape pomace and pecan nut shell UV-Vis spectra (Figure 10B,C) showed a band with a maximum at circa 280 nm, probably related to the presence of condensed tannins (Figure S7A), which was more intense for the untreated materials, suggesting significant degradation of these components perfectly in line with the results of the antioxidant assays, showing that the hydrolytic treatment led to a decrease of the antioxidant properties for both samples (Figure 1). This hypothesis was corroborated by the similar behavior exhibited by condensed quebracho tannins (Figure S7).

#### 3.4.3. Chemical Degradation Analysis 

In order to investigate how the hydrolytic treatment affected the structure of the main, non-extractable phenolic components of the different agri-food byproducts, these latter were subjected to chemical degradation treatments commonly used for the qualitative and quantitative analysis of phenolic polymers. These involved alkaline hydrogen peroxide degradation, alkali fusion and acid degradation. The first two methods are commonly employed to analyze insoluble and structurally complex phenolic polymers such as melanin pigments and are based on the identification of chromatographable, low-molecular weight markers, deriving from oxidative breakdown of the polymer [35,80]. As to the acid degradation method, this has been proposed as a validated approach for the characterization of extractable and nonextractable ellagitannins in plant materials [81]. 

**Figure 11:**
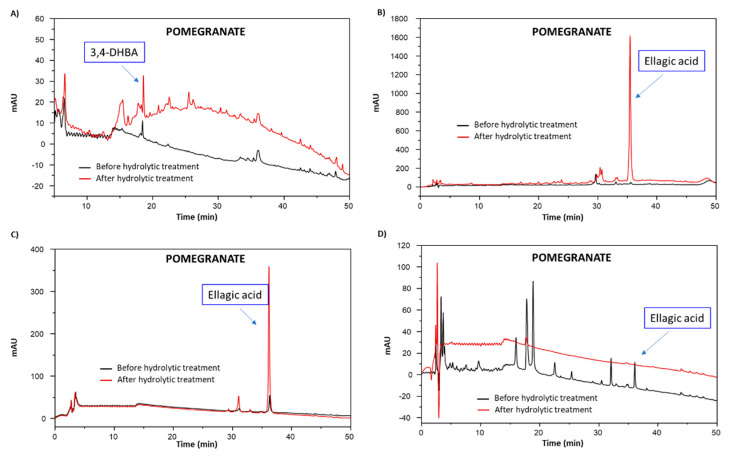
HPLC elution profiles of the chemical degradation mixtures of pomegranate. (**A**) Alkaline hydrogen peroxide degradation mixture. (**B**) Alkali fusion mixture. (**C**) Solid residue from the acid degradation mixture. (**D**) Supernatant from the acid degradation mixture.

The elutographic profiles of the chemical degradation mixtures obtained for the different agri-food byproducts before and after hydrolytic treatment are shown in Figures S8–S12. In most cases, the hydrolyzed materials showed chromatographic profiles characterized by more intense and/or a greater number of peaks than the untreated samples, suggesting again the occurrence of breaking/cleavage processes at the expenses of high molecular weight components, leading to the release of small units more susceptible to the attack by the chemical degradation agents and responsible for the improved antioxidant properties. 3,4-dihydroyxbenzoic acid (3,4-DHBA), 4-hydroxybenzoic acid (4-HBA) and gallic acid were identified as the main chemical degradation products by comparison of the chromatographic properties with those of reference standards. As an example, the HPLC profile of the alkaline hydrogen peroxide degradation mixture of untreated pomegranate waste showed a single peak eluted at 18 min and identified as 3,4-DHBA, which was detected in higher amounts in the case of the hydrolyzed sample, together with a complex mixture of unidentified compounds (Figure 11A). Furthermore, the HPLC profile of the alkali fusion mixture of the sample subjected to the hydrolytic treatment was characterized by the presence of ellagic acid as a major peak, which was practically absent in the mixture from untreated pomegranate (Figure 11B). Ellagic acid represented the main component also of the solid residue deriving from the acid degradation mixture, with an intensity about seven-fold higher than that obtained from the untreated pomegranate (Figure 11C). On the other hand, the HPLC profile of the supernatant from the acid degradation mixture of untreated pomegranate showed a series of peaks eluted at around 18–23 min (Figure 11D), likely due to partially hydrolyzed ellagitannins, which were found to be absent in the HPLC profile of pomegranate waste after the hydrolytic treatment, probably because this treatment had already removed these components. 

Pecan nut shell and grape pomace were once again an exception, with HPLC profiles related to the hydrolyzed materials showing less intense peaks than those obtained for the untreated samples, as particularly evident in the case of the supernatants obtained after acid degradation (Figures S11 and S12). These results further supported the hypothesis that the hydrolytic treatment led to a decrease of the low molecular weight, more accessible, soluble components, likely mainly involved in the antioxidant action.

Figure S13 sums up the possible chemical degradation pathways operating under the different reaction conditions adopted. 

## 4. Conclusions

**Figure 12:**
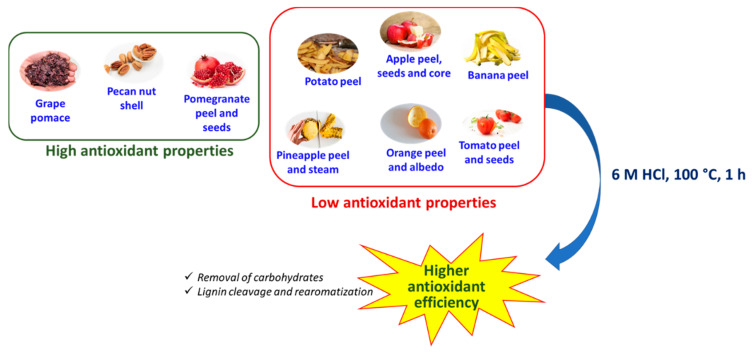
Overview of the antioxidant properties of agri-food byproducts and of the boosting effects of hydrolytic treatments.

The present paper reports the results of a systematic investigation of the antioxidant properties of a series of industrially relevant agri-food wastes, generally recognized as cheap and largely available sources of phenolic compounds such as lignins and tannins. The major role played by several factors as determinants of the antioxidant efficiency involving not only the amount and kind of the phenolic components present in each material, but also their solubility and accessibility, has been highlighted. The effects of an acid hydrolytic treatment previously applied to other agri-food wastes to improve the antioxidant properties were also evaluated. By comparing effects on both natural and bioinspired model systems, it appears that the marked enhancement of the antioxidant activity observed in most cases following acidic treatment is due both to removal of inert components (mainly carbohydrates), that hindered interaction of the phenolic-rich fractions with oxidants, and to structural modifications of the active phenolic fraction enhancing H-atom and electron donor properties of the lignin and/or hydrolyzable tannin-rich materials. Conversely, the marked decrease further to the same hydrolytic treatment in the antioxidant capacity of agri-food byproducts rich in condensed tannins such as grape pomace and pecan nut shell, can be attributed to partial loss of the low molecular weight, more accessible, antioxidant components (Figure 12). 

Overall, these results would put the basis for a rational exploitation of agri-food byproducts, in view of their antioxidant potential, boosted when appropriate by a hydrolytic treatment, e.g., as functional additives in active packaging. Future studies aimed at evaluation of other properties of biological relevance are desirable to broaden the application fields of these materials, e.g., as food supplements. In this context, the low cytotoxicity exhibited by related agri-food wastes even after the hydrolytic activation treatment provides a promising basis to further investigate this issue. The clever combination of hydrolyzed agri-food byproducts with oxidative manipulations in the presence of quinone-trapping and adhesion-promoting diamines, such as hexamethylenediamine, is also under investigation as a viable opportunity toward the development of natural product-based, mussel-inspired antioxidant coatings for surface functionalization and biomedical applications.

## Figures and Tables

**Table 1:** Antioxidant properties of agri-food byproducts. ^1^.

Sample	EC_50_ (mg/mL) ^2^ (DPPH Assay)	Trolox Eqs (FRAP Assay)	TPC (mg of Gallic Acid/g of Sample) (Folin-Ciocalteu Assay)
Pomegranate peel and seeds	0.29 ± 0.04	0.044 ± 0.009	125.6 ± 0. 9
Apple peel, seeds and core	>1.5	0.0006 ± 0.0007	23 ± 1
Orange peel and albedo	>1.5	0.0008 ± 0.0002	17 ± 1
Tomato peel and seeds	> 1.5	0.00046 ± 0.00001	25 ± 3
Potato peel	> 1.5	0.00023 ± 0.00001	27.7 ± 0.8
Banana peel	> 1.5	0.0044 ± 0.0002	20 ± 2
Pineapple peel and stem	> 1.5	0.00141 ± 0.00004	6.4 ± 0.3
Grape pomace	0.49 ± 0.05	0.0116 ± 0.0007	124 ± 5
Pecan nut shell	0.137 ± 0.005	0.061 ± 0.002	82 ± 4
Trolox	0.011 ± 0.001	-	

^1^ Reported are the mean ± SD values of at least three experiments. ^2^ EC_50_ is the dose of the material at which a 50% DPPH reduction is observed. FRAP: ferric reducing/antioxidant power; DPPH: 2,2-Diphenyl-1-picrylhydrazyl.

**Table 2:** Recovery yields of hydrolyzed waste materials. ^1^

Sample	Yields (*w*/*w*)
Pomegranate peel and seeds	10%
Apple peel, seeds and core	2%
Orange peel and albedo	9%
Tomato peel and seeds	12%
Potato peel	2%
Banana peel	7%
Pineapple peel and stem	20%
Grape pomace	30%
Pecan nut shell	45%

^1^ Reported are the mean values of at least three experiments (SD ≤ 5%).

## Supplementary Materials

The following are available online at https://www.mdpi.com/2076-3921/9/5/438/s1, Figure S1. UV-Vis spectra of PolyCAF and PolyFER; Figure S2. CPNQS and CPPI NMR spectra of PolyFER; Figure S3. ATR-FTIR spectra of selected agri-food byproducts; Figure S4. UV-Vis spectra of the extractable fraction of selected agri-food byproducts; Figure S5. HPLC profile of the extractable fraction of pomegranate waste; Figure S6. UV-Vis spectra and HPLC profiles of the extractable fraction of chestnut tannins; Figure S7. UV-Vis spectra and HPLC profiles of the extractable fraction of quebracho tannins; Figure S8. Alkali degradation and alkali fusion mixture HPLC profiles of apple, orange, tomato and potato wastes; Figure S9. Alkali degradation and alkali fusion mixture HPLC profiles of pineapple, grape pomace and banana waste; Figure S10. Acid degradation mixture HPLC profiles of apple, orange, tomato and potato wastes; Figure S11. Acid degradation mixture HPLC profiles of pineapple, grape pomace and banana waste; Figure S12. Alkali degradation, alkali fusion and acid degradation mixture HPLC profiles of pecan nut shell; Figure S13. Possible chemical degradation pathways operating under alkaline and acid conditions.
