# Supplementary material for: Antioxidant Properties of Agri-Food Byproducts and Specific Boosting Effects of Hydrolytic Treatments
Source: Antioxidants (Basel). 2020 May 18;9(5):438. doi: 10.3390/antiox9050438 (PMC7278820; doi:10.3390/antiox9050438)
Supplement: Supplementary file 1 [file antioxidants-09-00438-s001.pdf]

Antioxidant Properties of Agri-food Byproducts and Specific Boosting Effects of Hydrolytic Treatments

Federica Moccia [1], Sarai Agustin-Salazar [2], Luisella Verotta [3], Enrico Caneva [4], Samuele Giovando [5], Gerardino D'Errico [1,6], Lucia Panzella [1,*], Marco d'Ischia [1] and Alessandra Napolitano [1]

[1] Department of Chemical Sciences, University of Naples "Federico II", Via Cintia 4, I-80126 Naples, Italy
[2] Departamento de Ingeniería Química y Metalurgía, Universidad de Sonora, Del Conocimiento, Centro, 83000 Hermosillo, México
[3] Dipartimento di Scienze e Politiche Ambientali, Università degli Studi di Milano, Via G. Celoria 2, I-20133 Milan, Italy
[4] Unitech COSPECT, Direzione servizi per la Ricerca, Università degli Studi di Milano, Via C. Golgi 33, I-20133 Milan, Italy
[5] Centro Ricerche per la Chimica Fine Srl for Silvateam Spa, Via Torre 7, I-12080, San Michele Mondovì, CN, Italy
[6] CSGI - Consorzio Sistemi a Grande Interfase, Department of Chemistry, University of Florence, Via della Lastruccia 3, I-50019, Sesto Fiorentino (FI), Italy
* Correspondence: panzella@unina.it; Tel.: +39-081-674131

Table of contents **Page**

Figure S1. UV-vis spectra of PolyCAF and PolyFER. 1

Figure S2. CPNQS and CPPI NMR spectra of PolyFER. 2

Figure S3. ATR-FTIR spectra of selected agri-food byproducts. 3

Figure S4. UV-Vis spectra of the extractable fraction of selected agri-food byproducts. 4

Figure S5. HPLC profile of the extractable fraction of pomegranate waste. 5

Figure S6. UV-vis spectra and HPLC profiles of the extractable fraction of chestnut tannins. 6

Figure S7. UV-vis spectra and HPLC profiles of the extractable fraction of quebracho tannins. 7

Figure S8. Alkali degradation and alkali fusion mixture HPLC profiles of apple, orange, tomato and potato wastes. 8

Figure S9. Alkali degradation and alkali fusion mixture HPLC profiles of pineapple, grape pomace and banana waste. 9

Figure S10. Acid degradation mixture HPLC profiles of apple, orange, tomato and potato wastes. 10

Figure S11. Acid degradation mixture HPLC profiles of pineapple, grape pomace and banana waste. 11

Figure S12. Alkali degradation, alkali fusion and acid degradation mixture HPLC profiles of pecan nut shell. 12

Figure S13. Possible chemical degradation pathways operating under alkaline and acid conditions. 13

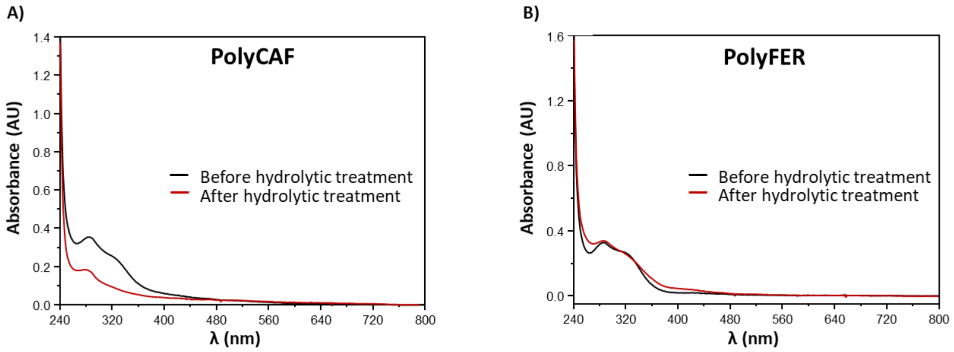

Figure S1. UV-Vis spectra in methanol at 0.01 mg/mL concentration (from dilution of a 0.33 mg/mL solution in DMSO) of (**A**) PolyCAF and (**B**) PolyFER before (black traces) and after (red traces) hydrolytic treatment.

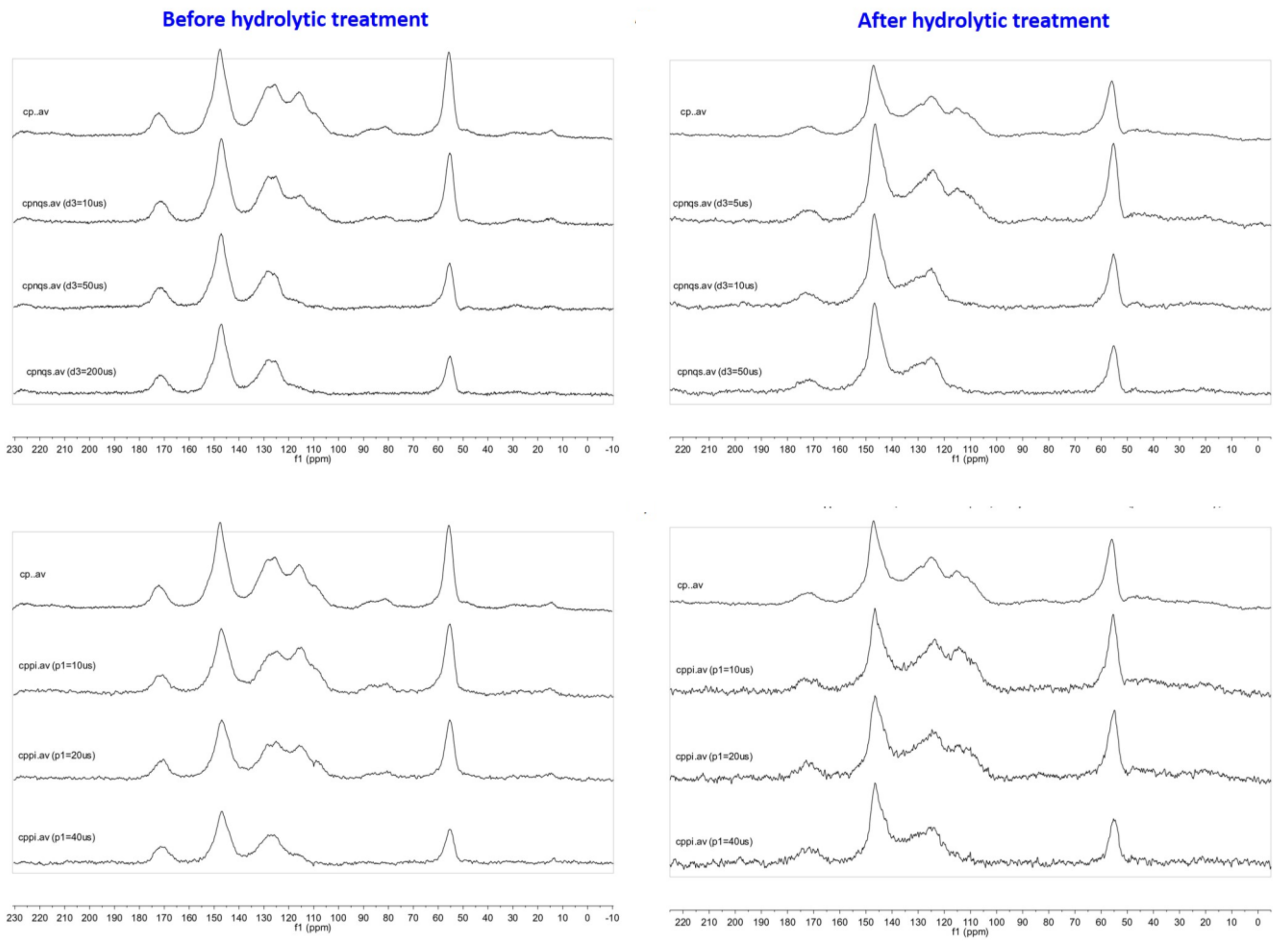

Figure S2. CP-MAS with rotor synchronized NQS (top) and CP editing experiment by phase inversion (CPPI) (bottom) ^{13}C NMR spectra of PolyFER before and after hydrolytic treatment.

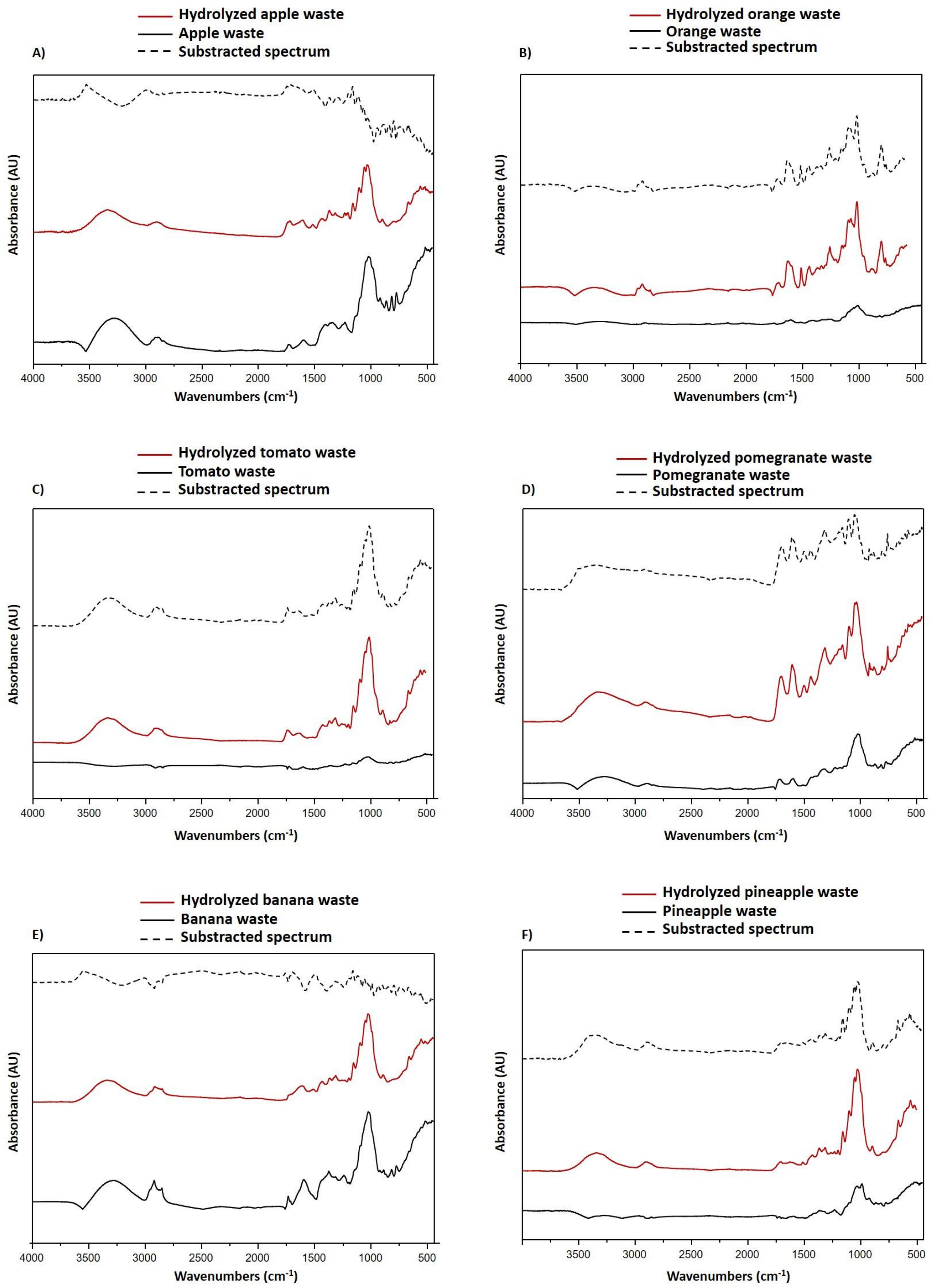

Figure S3. ATR-FTIR spectra of (**A**) apple waste, (**B**) orange waste, (**C**) tomato waste, (**D**) pomegranate waste, (**E**) banana waste, and (**F**) pineapple waste.

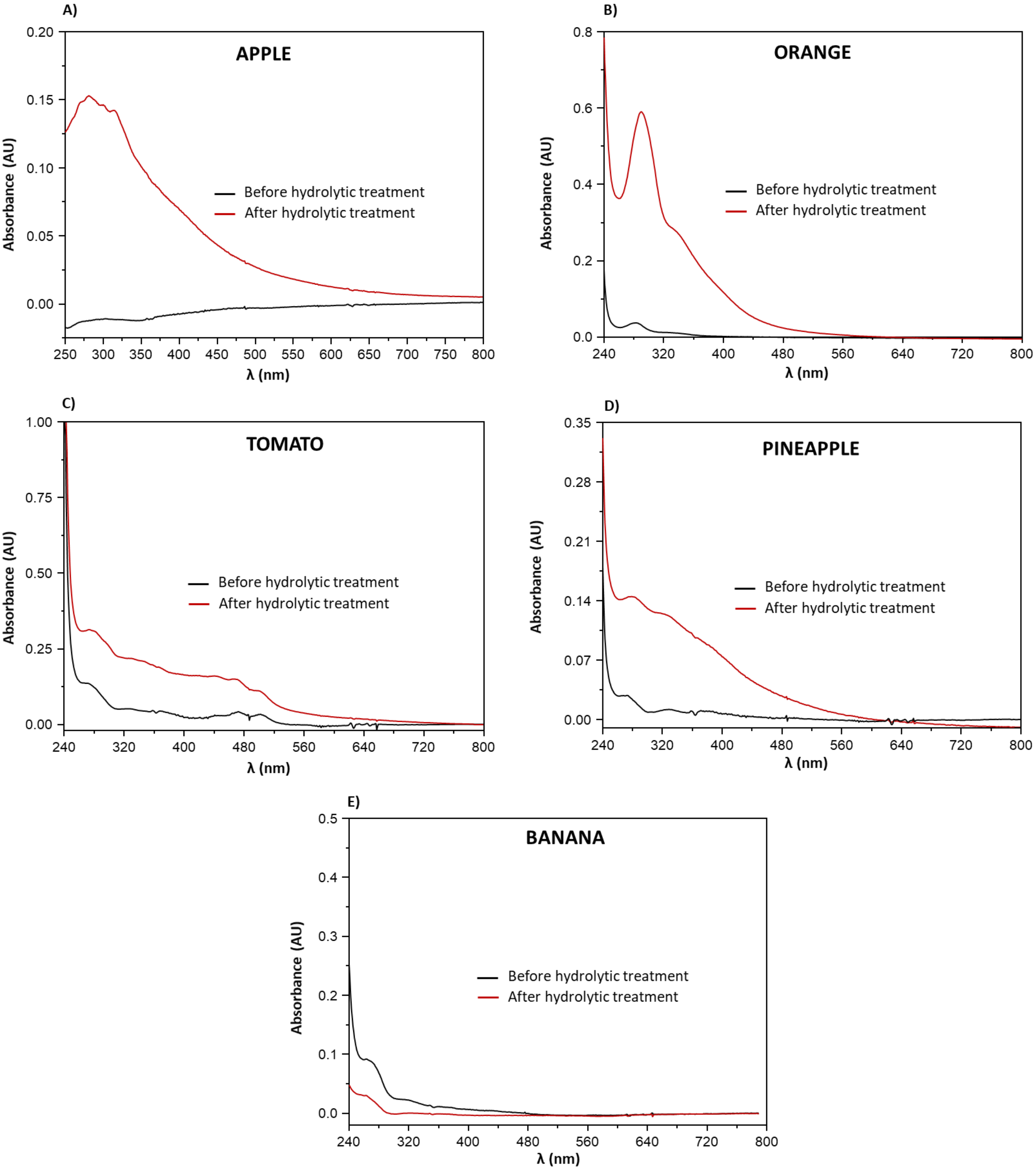

Figure S4. UV-Vis spectra of the DMSO-soluble fraction of (**A**) apple waste (0.2 mg/mL), (**B**) orange waste (0.2 mg/mL), (**C**) tomato waste (0.2 mg/mL), (**D**) pineapple waste, and (**E**) banana waste before and after the hydrolytic treatment. Concentrations refer to the starting dose of each agri-food waste in DMSO after proper dilution in methanol (see paragraph 2.11 in the main text).

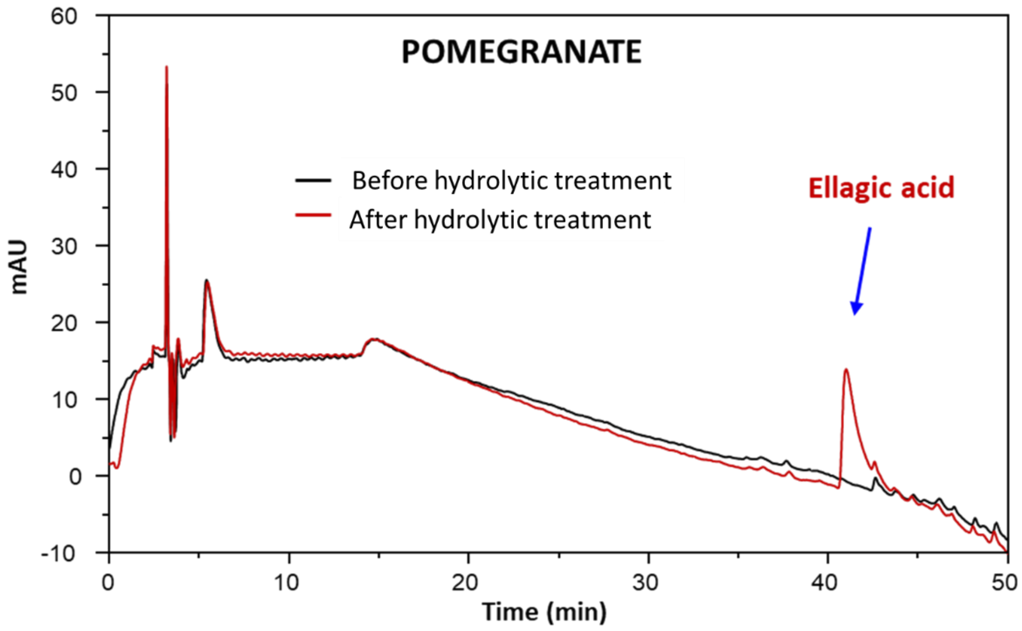

Figure S5. HPLC profile of the DMSO-extractable fraction of pomegranate waste before and after hydrolytic treatment.

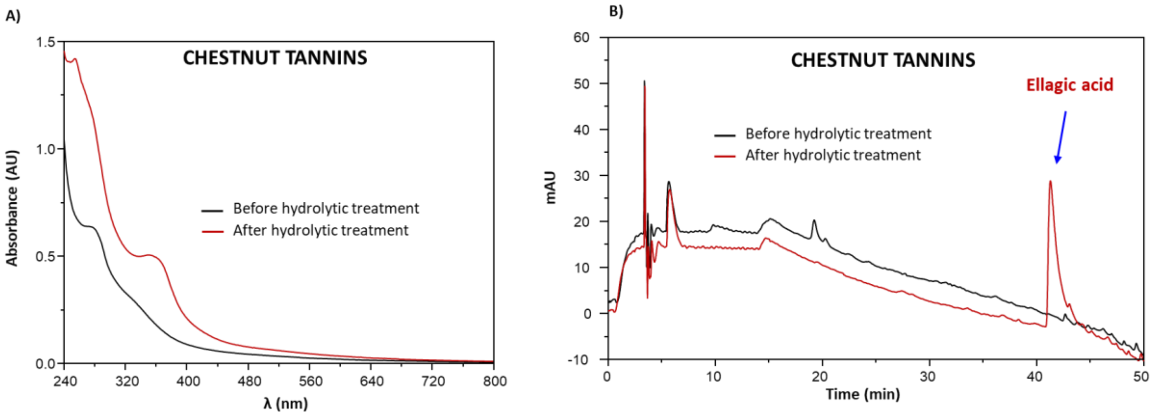

Figure S6. (**A**) UV-vis spectra (0.02 mg/mL) and (**B**) HPLC profiles of the DMSO-extractable fraction of chestnut tannins, before and after hydrolytic treatment.

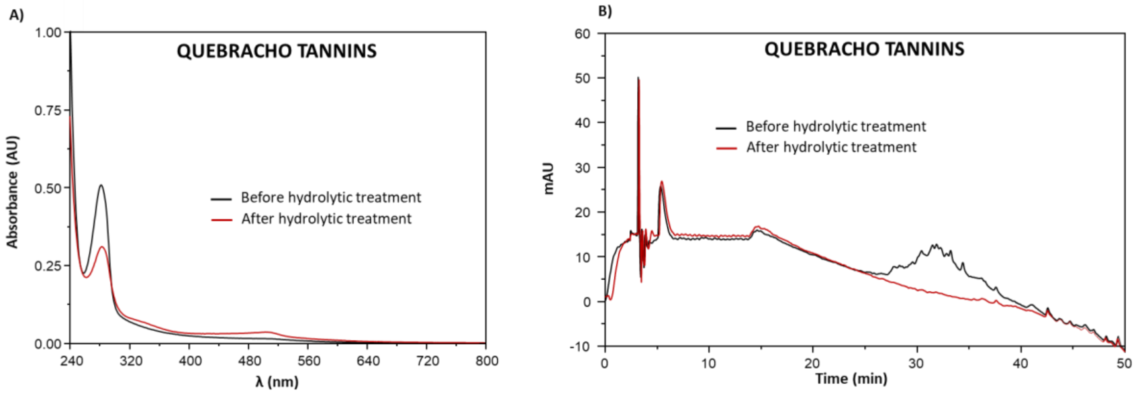

Figure S7. (**A**) UV-vis spectra (0.02 mg/mL) and (**B**) HPLC profiles of the DMSO-extractable fraction of quebracho tannins, before and after hydrolytic treatment.

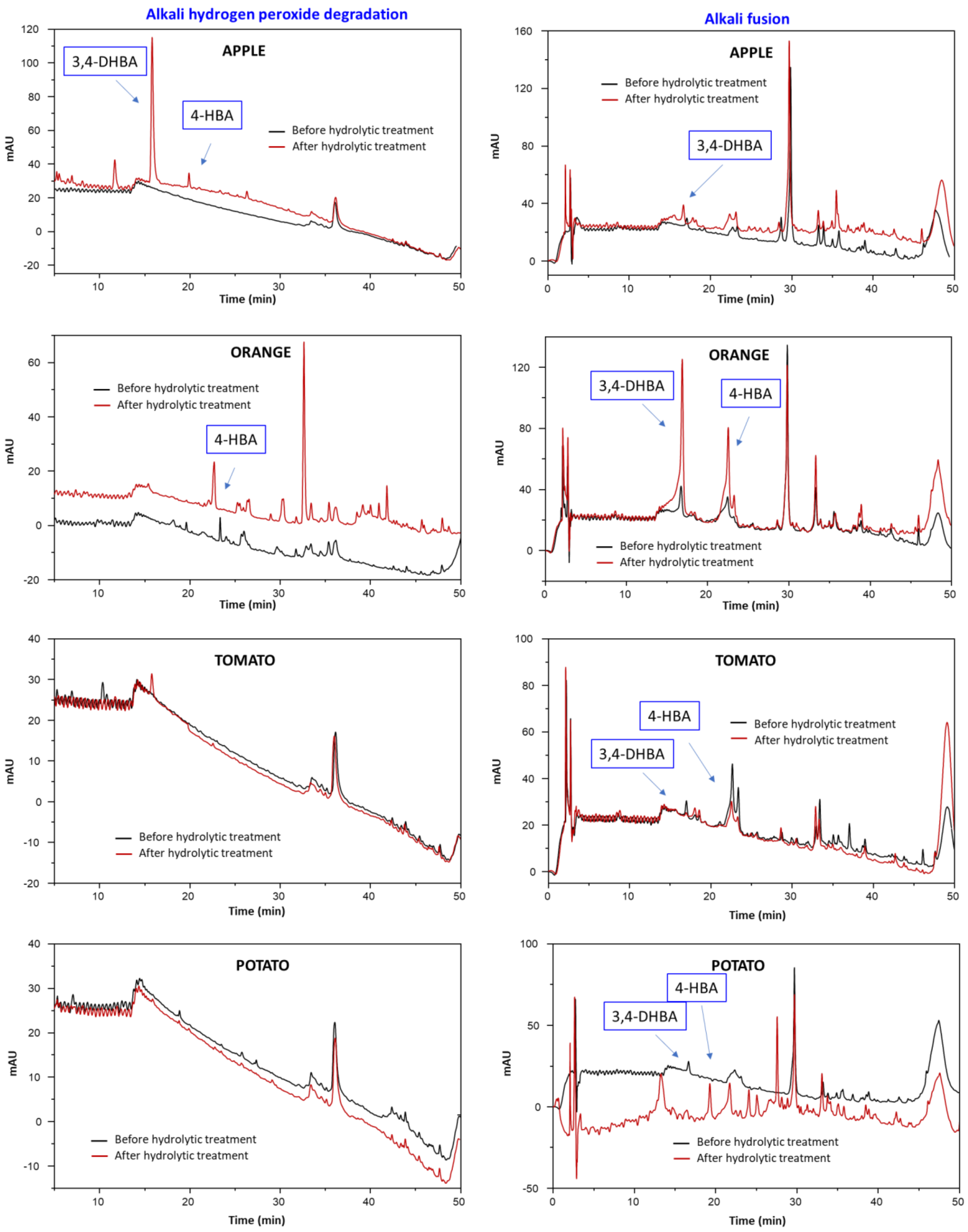

Figure S8. Alkali degradation and alkali fusion HPLC profiles of apple, orange, tomato and potato wastes before and after the hydrolytic treatment. 3,4-DHBA: dihydroybenzoic acid. 4-HBA: 4-hydroxybenzoic acid.

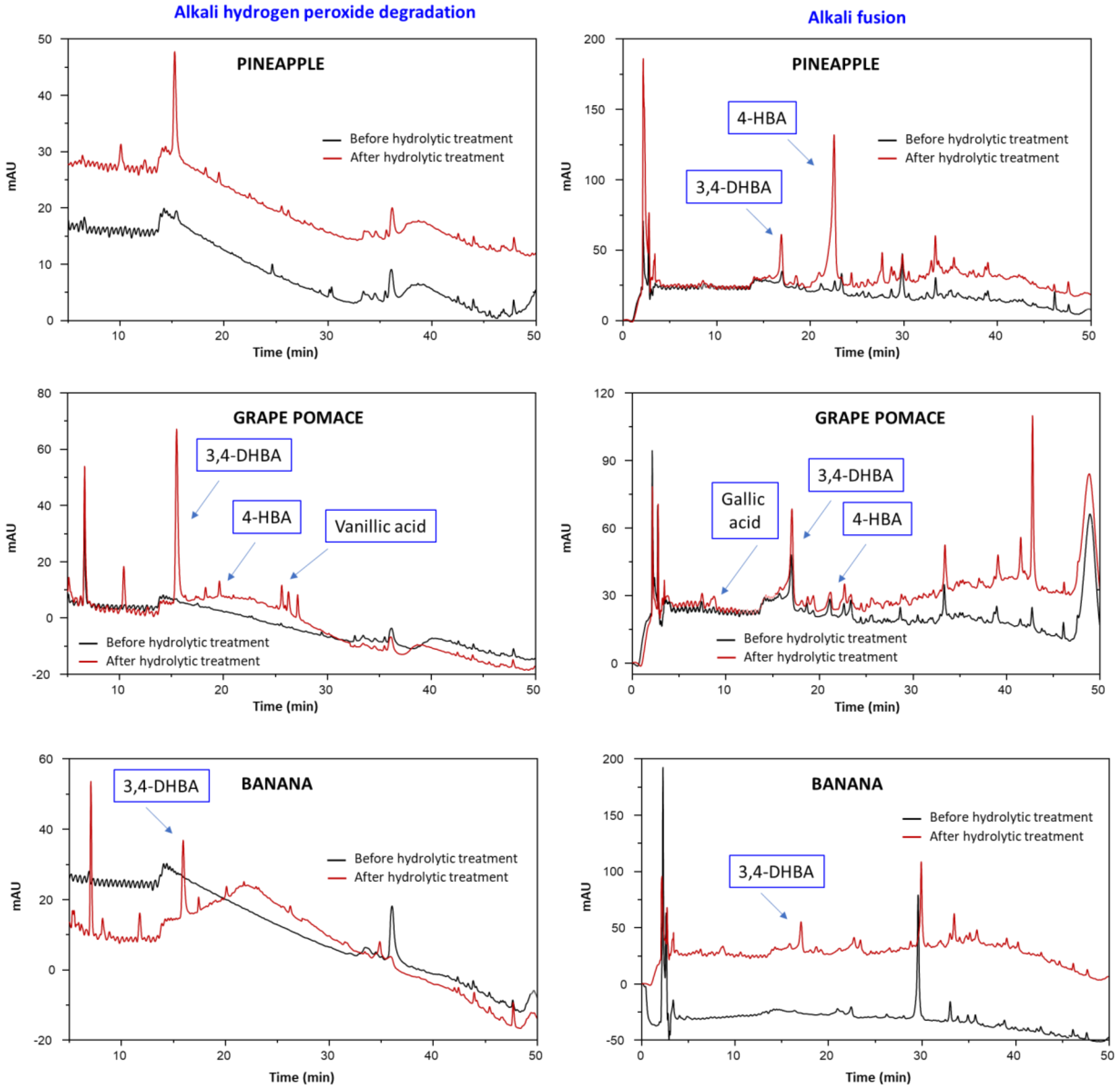

Figure S9. Alkali degradation and alkali fusion HPLC profiles of pineapple waste, grape pomace and banana waste before and after the hydrolytic treatment. 3,4-DHBA: dihydroybenzoic acid. 4-HBA: 4-hydroxybenzoic acid.

.

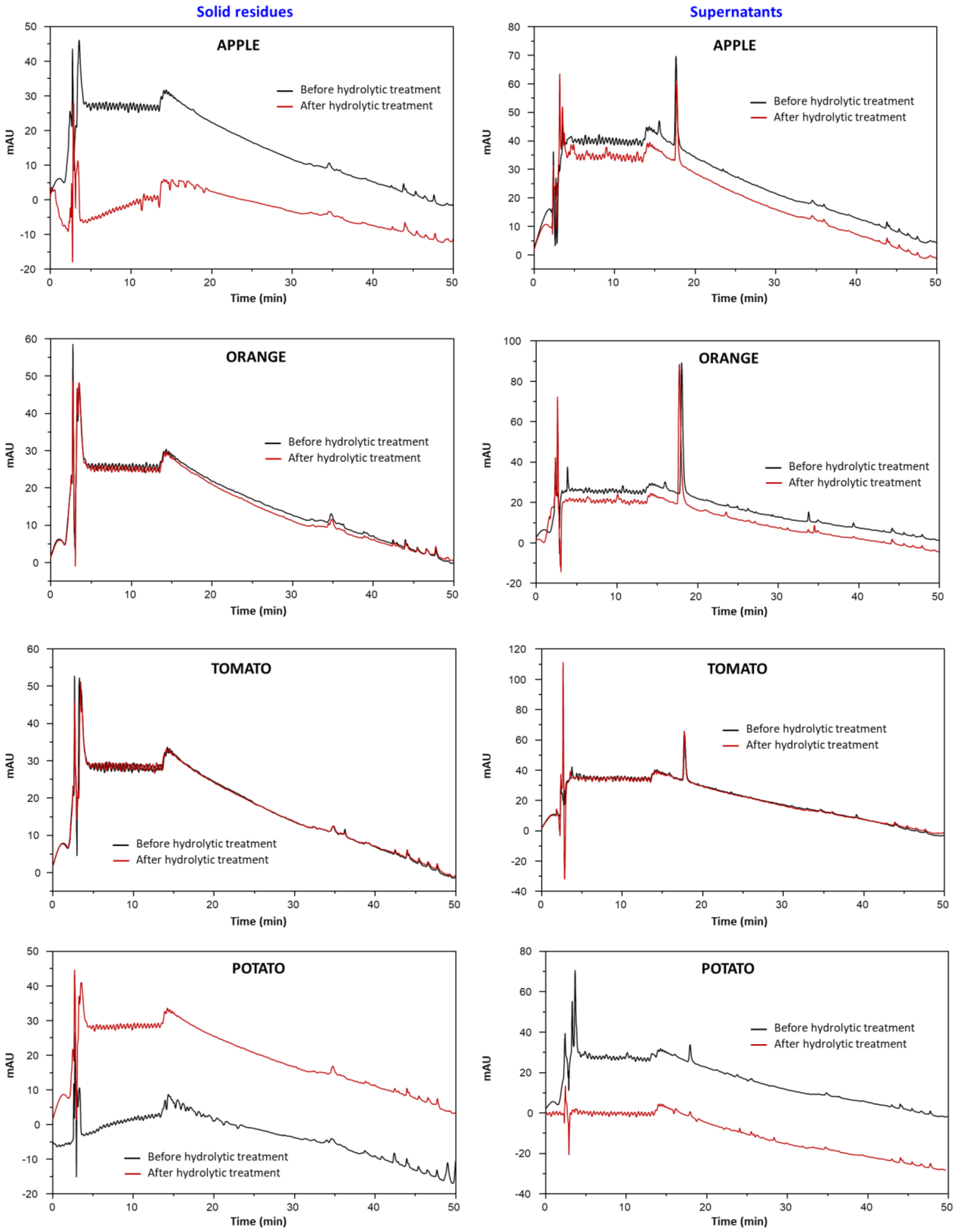

Figure S10. HPLC profiles of the supernatants and solid residues from acid degradation of apple, orange, tomato and potato wastes.

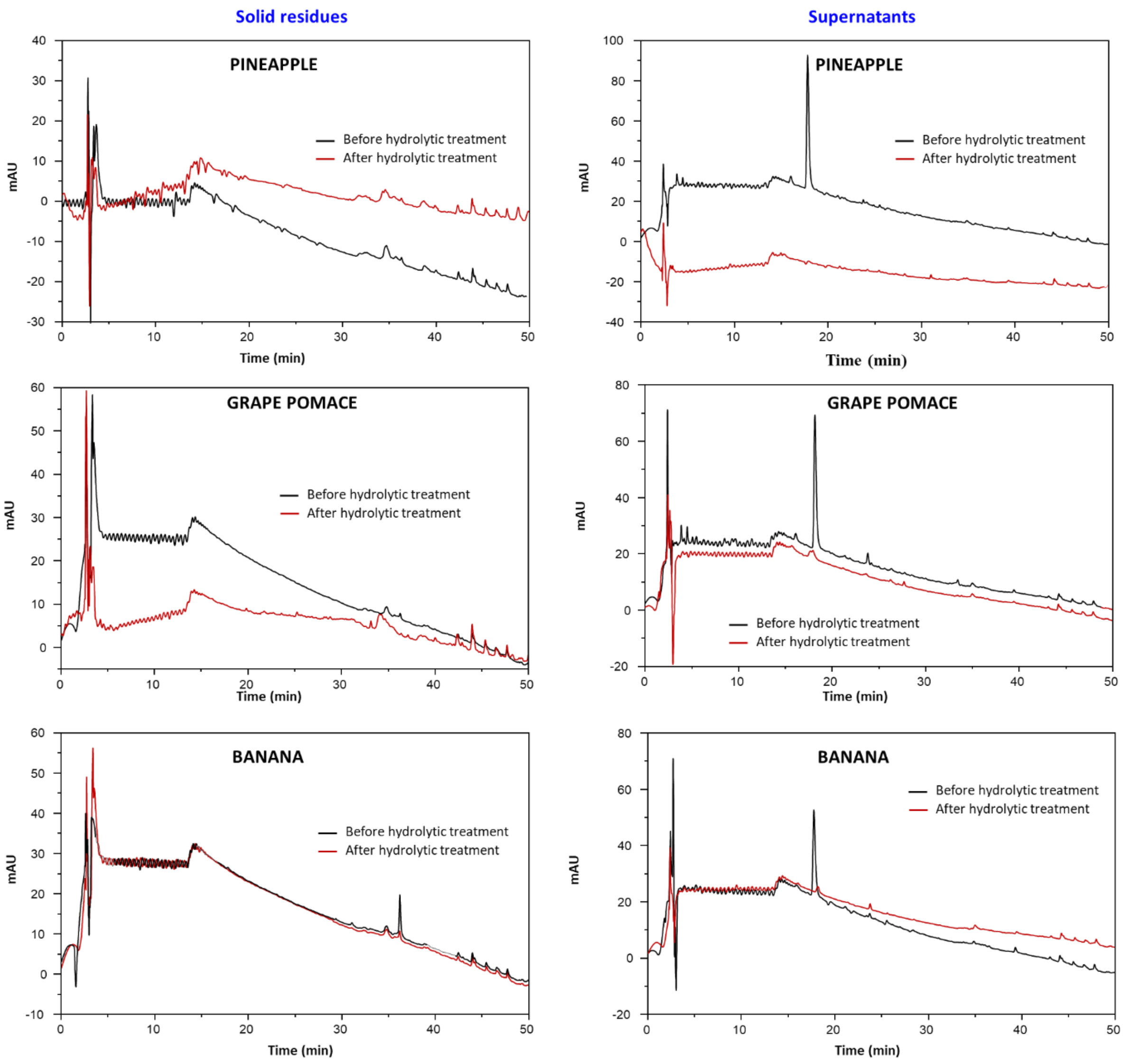

Figure S11. HPLC profiles of the supernatants and solid residues from acid degradation of pineapple waste, grape pomace and banana waste.

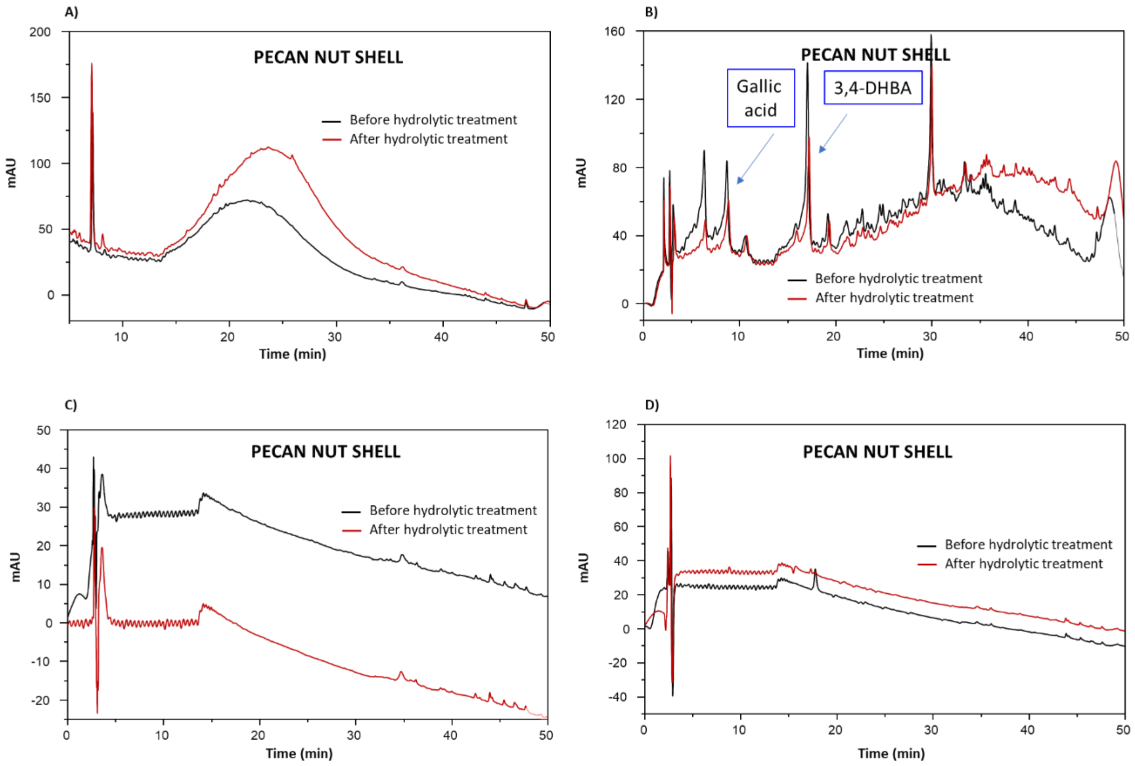

Figure S12. HPLC profiles of the chemical degradation mixtures of pecan nut shell. (**A**) Alkaline hydrogen peroxide degradation mixture. (**B**) Alkali fusion mixture. (**C**) Solid residue from the acid degradation mixture. (**D**) Supernatant form the acid degradation mixture.

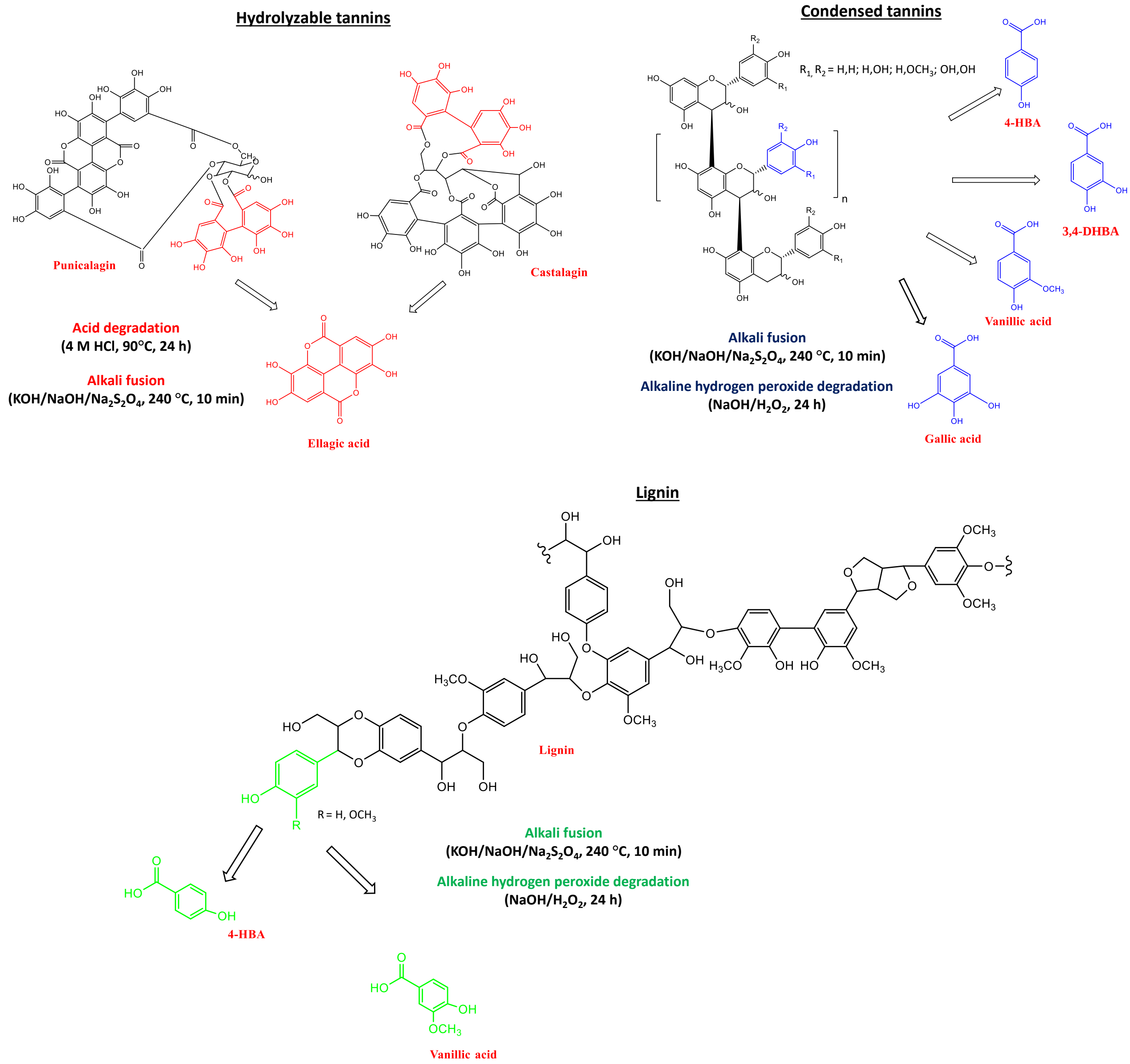

Figure S13. Possible chemical degradation pathways operating under alkaline and acid conditions.
